# Predicting RNA secondary structure via adaptive deep recurrent neural networks with energy-based filter

**DOI:** 10.1186/s12859-019-3258-7

**Published:** 2019-12-24

**Authors:** Weizhong Lu, Ye Tang, Hongjie Wu, Hongmei Huang, Qiming Fu, Jing Qiu, Haiou Li

**Affiliations:** 1 School of Electronic and Information Engineering, Suzhou University of Science and Technology, Suzhou, Jiang 215000 China; 2 Anhui Key Laboratory of Intelligent Building Energy Efficiency, Anhui Jianzhu University, Hefei, Anhui 230601 China

**Keywords:** RNA, Secondary structure prediction, Recurrent neural network, LSTM, Pseudoknots

## Abstract

**Background:**

RNA secondary structure prediction is an important issue in structural bioinformatics, and RNA pseudoknotted secondary structure prediction represents an NP-hard problem. Recently, many different machine-learning methods, Markov models, and neural networks have been employed for this problem, with encouraging results regarding their predictive accuracy; however, their performances are usually limited by the requirements of the learning model and over-fitting, which requires use of a fixed number of training features. Because most natural biological sequences have variable lengths, the sequences have to be truncated before the features are employed by the learning model, which not only leads to the loss of information but also destroys biological-sequence integrity.

**Results:**

To address this problem, we propose an adaptive sequence length based on deep-learning model and integrate an energy-based filter to remove the over-fitting base pairs.

**Conclusions:**

Comparative experiments conducted on an authoritative dataset RNA STRAND (RNA secondary STRucture and statistical Analysis Database) revealed a 12% higher accuracy relative to three currently used methods.

## Introduction

RNA is a carrier of genetic information, and its structure plays a crucial role in gene maturation, regulation, and function [1–3]. Studying the relationship between RNA function and structure and determining the form and frequency of RNA folding are important to reveal the role of RNA molecules in the life process [4–6]. The most common way to manipulate RNA structures algorithmically is to reduce them to base pairs (i.e., secondary structures) abstracted from the actual spatial arrangement of nucleotides. For a valid secondary structure, each base, *i*, can only interact with at most one other base, *j*, and form one base pair (*i, j*) [7, 8].

The secondary structure of an RNA molecule represents base-pair interactions that fundamentally determine overall structure [9–11]. Current studies of RNA molecular structure emphasize the difficulty of RNA secondary structure analysis [12, 13].

Pseudoknots are substructures of RNA secondary structure that describe crossed base pairs [(*i*, *j*) and (*k*, *l*)] in a sequence, where *i* < *k* < *j* < *l*. RNA secondary structure prediction in the absence of pseudoknots has been studied using dynamic programming algorithms described by Zuker [14] and Mathews [15, 16] and employing m-fold [17] and GT-fold [18]. From an algorithmic standpoint, RNA pseudoknotted secondary structure prediction represents an NP-hard optimization problem [19]; therefore, in order to reduce computational complexity, most algorithms ignore pseudoknots [7].

Common RNA secondary structure prediction models mainly include thermodynamic models, homology comparison models, and statistical-learning models [20]. The thermodynamic model assumes that RNA molecules are subject to the laws of thermodynamics, and that RNA structures with a lower free energy are more stable. Therefore, from all possible secondary structures, that with smallest free energy represents the optimal predicted result. A homologous comparison model searches for commonly mutated base pairs in sequences from the same source, although homologous sequences need to be additionally provided as input [21]. The statistical-learning model can predict the regularity of known RNA structures through machine learning and other methods, with accuracies that can potentially exceed those associated with the method targeting the minimum free energy.

After translating the RNA secondary structure-prediction problem into a classification problem of base pairings in the sequence by using machine-learning algorithms, computational complexity can be reduced, but only to a certain extent. However, there remain two difficulties. First, the existence of pseudoknots makes the folding of RNA sequences more complicated; therefore, bases cannot be distinguished using only three categories of “unpaired bases”, “paired bases near the head”. and “paired bases near the end”. The E-NSSE method, which divides bases into five categories, can only predict plane pseudoknots, but cannot predict complex structures involving nonplanar pseudoknots. Second, a machine-learning model using a fixed-sized vector as the input feature is unsuitable for processing sequence-type data and cannot process RNA sequences of variable length.

As a research hotspot in the field of machine learning, deep learning can mine deeper hidden features from data [22–24]. A recurrent neural network is a sequence-oriented neural-network model for deep learning that displays excellent performance in natural-language processing, image recognition, and speech recognition [25, 26].

However, common deep neural-network models are restricted to features with a fixed shape and, therefore, cannot model RNA primary structures with variable sequence lengths. Here, we applied a long short-term memory (LSTM) network to establish a secondary structure-prediction method that is adaptable to RNA sequences of variable length. A previous study by Mathews [27] showed that a higher base-pairing probability calculated by the partition function resulted in a greater the probability of its appearance in the real structure. Therefore, the type of base and the output of its partition function was selected as the feature of the base. Additionally, we introduced a mask vector to eliminate the effect of the extended sequence on the model, which allowed the model to process variable length RNA primary sequences. A weight vector was used to dynamically regulate the proportion of the loss function associated with each base in the total loss function of the sequence, thereby alleviating the unbalanced distribution of the samples. However, there may be some conflicting predicted bases in the predicted result of LSTM, for example, the *i*-th base is predicted to be paired with the *j*-th base, but the *j*-th base is predicted to be an unpaired base or paired with another base. To solve the problem, a energy-based filter is also proposed to filter the conflicting predicted result of LSTM.

## Methods

### RNA secondary structure prediction

A, G, C, U are four different bases in RNA molecules, several bases are arranged in order to form the primary structure of RNA [28, 29]. The primary structure of an RNA sequence *S* consisting of n bases can be expressed as *S* = *s*_1_, *s*_2_, ... *s*_n_, where s_1_ is the base near the 5′ side, *s*_*n*_ is the base near the 3′ side, *s*_*i*_ is the *i*-th base in sequence *S* and *s*_*i*_∈{A, G, C, U}.

RNA secondary structure prediction problem, with the purpose of calculating the pairing results *y*_*i*_ of each base *s*_*i*_ in sequence *S* when the primary structure of *S* is known, is a classification problem. According to different categories of classification, RNA secondary structure prediction problems can be divided into the following categories:

1. Two categories classification: pairing results consist of the category of paired base (*y*_*i*_ = 1) and the category of not paired base (*y*_*i*_ = 0).
2. Three categories classification: pairing results include the category of paired base near 5′ side (*y*_*i*_ = 1), the category of paired base near 3′ side (*y*_*i*_ = 2) and the category of not paired base (*y*_*i*_ = 0).
3. Multi-category classification: for a sequence with pseudoknots, pairing results *y*_*i*_ = *j*(*j* = 0,1 … n) means the *i*-th base is paired with the *j*-th base if *j* > 0, otherwise the *i*-th base is not paired.

This paper focus on (1, 3).

### Adaptive LSTM with energy-based model

**Fig. 1:**
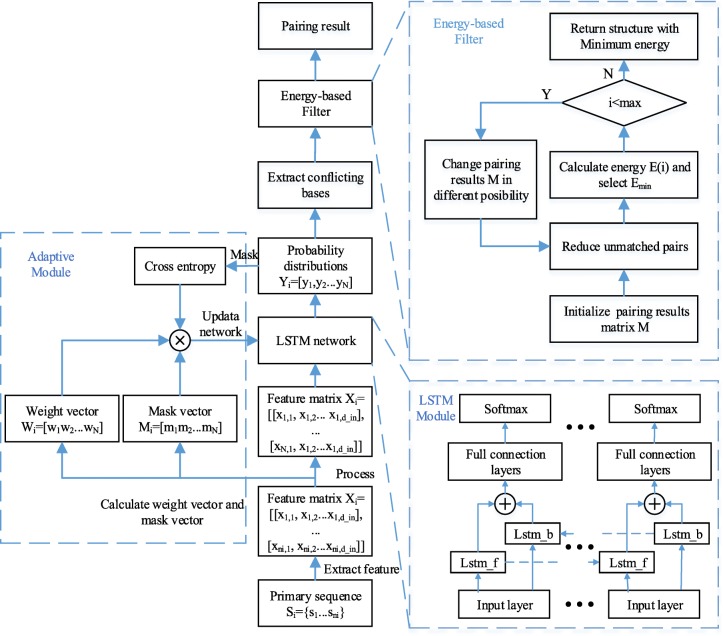
Framework $\mathrm{X}\in {\mathrm{R}}^{{\mathrm{I}}_{{\mathrm{l}}_1}^{\mathrm{author}}\times {\mathrm{I}}_{{\mathrm{l}}_2}^{\mathrm{paper}}\times {\mathrm{I}}_{{\mathrm{l}}_3}^{\mathrm{term}}}$ of the Adaptive-LSTM with filter

The scheme of a RNA secondary structure-prediction model based on Adaptive LSTM and energy-based filter is shown in Fig. 1.

The input feature matrix of a sequence is a two-dimensional tensor *X*. *X* [*i, j*] represents the *j*-th feature of the i-th base, the *N* + 5 dimensional vector *X* [*i*] is the input feature vector of a base, the first element of *x* [*i*] is the frequency of the base in the sequence, the second to 5th elements represent the type of base, replacing A,U,C,G and unknown type by [0,0,0,1], [0,0,1,0], [0,1,0,0], [1,0,0,0] and [0,0,0,0]. The last N elements are the outputs of the partition function. The output label is set as the multi-category classification in section 2.1.

This model capable of predicting RNA pseudoknots comprises adaptive module, LSTM module and energy-based filter module.

LSTM module consists of an input layer, an LSTM layer, fully connected layers, and a softmax layer. The input layer maps the input features into higher dimensional feature vectors and inputs them into the forward LSTM unit lstm_f and the backward LSTM unit lstm_b in the LSTM layer. After splitting the outputs of the forward LSTM and the backward LSTM, they are input into the back-propagation neural network comprising the full connection layer and the output layer for classification. The fully connected layers use tanh as an activation function, and a dropout layer was added to improve fitting to the test data. The output layer uses softmax as an activation function and converts the output of the fully connected layer into a probability distribution vector that represents the probability that each base in the sequence belongs to each output category.

The adaptive module is used to make the model handle sequences with variable length and reduce the impact from imbalanced samples.

The energy-based filter module is used to filter the over-fitting pairs by selecting the structure with lower free energy. In the output of LSTM, each base s_i_, of the sequence has an independently predicted category *y*_*i*_, representing the most likely base index to be paired with. However, it is possible that some predicted results could have been duplicated or might have shown inconsistencies. For example, the *i*-th base was predicted as being paired with the *j*-th base (*y*_*i*_ = *j*), but the base with which the *j*-th base was predicted to be paired with was not the *i*-th base (*y*_*i*_ ≠ *j*). The energy-based filter correct some of these conflicting bases to make the predicted structure conform to the principle of base pairing and have lower free energy.

### Adaptive LSTM

A mask vector was introduced to enable the model to effectively process sequences of different length and to ensure that the extended base will not affect the normal training of the model.

Let the maximum length of the sequence the model can accept be *N*. For the *k*-th sequence, its length is *n*_*k*_, and the original sequence features comprising its feature of *n*_*k*_ bases are *x*(1), *x* (2) ... *x* (*n*_*k*_). Expand the sequence feature by setting the features of the *n*_*k*_ + 1-th to *N*-th base, *x* (*n*_*k*_ + 1), *x* (*n*_*k*_ + 2)...*x*(*N*), with an arbitrary value, so that all lengths of all sequences can be unified into a fixed value, *N*. An *N*-dimensional mask vector, M_k_, is generated (*M*_*k*_ = [*M*_*k*_(*i*) | *i* = 1, 2 ... *N*], *M*_*k*_(*i*) = 1) when *i* ≤ *n*_*k*_, and *M*_*k*_(*i*) = 0 when *n*_*k*_ < *i* ≤ *N.* The mask vector is used to distinguish between the original and extended parts of a sequence, and the new sequence represents the input to the LSTM network. Therefore, the cross-entropy loss function of this sequence is calculated as follows:

1 $$ {L}_k\left(y,{y}^{\hbox{'}}\right)=\frac{1}{n}\sum \limits_{i=1}^n{M}_k(i)\sum \limits_{j=0}^m{y}^{\hbox{'}}\left[i,j\right]\log \left(y\left[i,j\right]\right) $$

where *n* is the length of the sequence, m is the number of categories, the two-dimensional arrays *y* and *y’* represent the prediction result and the real label, respectively, and *y* [*i, j*] indicates the probability that the *i*-th base belongs to the *j*-th category.

When calculating the cross-entropy of the *i*-th base in the *k*-th sequence, *M*_*k*_(*i*) will appears as a product in the calculation of the gradient formula. When the *i*-th base is from the original sequence, (*i* ≤ *n*_*k*_) [*M*_*k*_(i) = 1], the gradient value will not be changed by *M*_*k*_(*i*), and the network weight will be updated similar to that in a conventional method. When the base is not found in the original sequence (*n*_*k*_ < *i* ≤ *N*) [*M*_*k*_(*i*) = 0], the gradient value will be 0, and the network weight will not be updated. Therefore, the invalid prediction by the model of extended bases will not affect the update of the model.

### Dynamic weighting method

Among the two categories used for classification of RNA secondary structure prediction, the ratio of the number of bases belonging to the paired and unpaired categories ~ 6:4 in the RNA STRAND dataset; however, in the multi-category classification problem, the ratio is ~ 6/*n*:6/*n*: …: 6/*n*:4. As n increases, there will be an uneven distribution of samples, which might lead a model to predict all bases as in the unpaired category, because the number of bases belonging to that category in the real structure is much larger than that of any other categories.

To address this problem, a dynamic weighting method was added to the model. For the *k*-th sequence, a weight vector, *W*_*k*_, was generated. If the *i*-th base is a paired base in the real structure, the value of *W*_*k*_(*i*) equals the number of unpaired bases in the sequence; otherwise, the value of *W*_*k*_(*i*) is 1. After adding the dynamic weighting method, the loss function of the *k*-th sequence is as follows:

$$ {L}_k\left(y,{y}^{\hbox{'}}\right)=\frac{1}{w}\sum \limits_{i=1}^n{W}_k(i){M}_k(i)\sum \limits_{j=0}^m{y}^{\hbox{'}}\left[i,j\right]\log \left(\mathrm{y}\left[i,j\right]\right) $$

$$ \mathrm{m}=\sum \limits_{k=1}^N{M}_k,\mathrm{w}=\sum \limits_{k=1}^N{W}_k\cdot {M}_k, $$

2 $$ {W}_k=\left\{{\displaystyle \begin{array}{cc}1& {y}^{\hbox{'}}\left[i,0\right]\ne 1\\ {}\sum \limits_{i=1}^n{y}^{\hbox{'}}\left[i,0\right]& {y}^{\hbox{'}}\left[i,0\right]=1\end{array}}\right. $$

### Energy-based filter

As the result of translating the RNA secondary structure-prediction problem into a classification problem of base pairings, there exist some conflicting pairing result in the output of LSTM. The energy-based filter is used to deal with this problem.

In laws of thermodynamics, RNA structures with a lower free energy are more stable [16], so the energy-based is used to randomly change the label of conflicting base pairings according to the free energy of the structure to make the structure more likely to its real structure.

According to the Watson-Crick base complementary pairing principle [23], each base *s*(*i*), can only interact with at most one other base, *s*(*j*), to form one base pair (*s*(*i*), *s*(*j*)) and {*s*(*i*), *s*(*j*)} ∈{{A,U},{C,G},{G,U}}. As a result, the predicted result of *i*-th base *y*(*i*) can be reserved if the two following conditions are met:

1. *y*(*y*(*i*))=*i*
2. (*s*(*i*), *s*(*y*(*i*))) is in {(A, U), (U, A), (C, G), (G, C), (G, U), (U, G)}

If these conditions are not met, *y*(*i*) should be set as 0 to classify the *i*-th base as unpaired base.

By setting all the conflicting bases to unpaired bases, it may incorrectly turn false positive samples into false negative samples, energy-based filter is improved from reducing unmatched pairs to filter pairing results by the free energy of secondary structure. The flow chart of energy-based filter is shown in the right upper of Fig. 1 and the energy-based filter algorithm is as follows:

In Extract_bases(*y*), bases with conflicting pairing result will be extracted from y (the predicted result of Adaptive LSTM) into conflicting base list *S*(*i*) and its index *Id*(*i*), *i*∈{0,1 … *m*^− 1^}, *S*(*i*), *Id*(i)∈{1,2 … *k*}, where m is the number of conflicting bases, *k* is the length of the primary sequence, *i* is the index of each base in the conflicting bases list, *Id*(i) is the index of the *i*-th base in its primary sequence, *S*(*i*) represents the predicted result of Adaptive LSTM of *i*-th conflicting base (the *Id*(*i*)-th base in its primary sequence).

The pairing result matrix *M*_*m*x*n*_, denoting *n* pairing results, is randomly initialized with 0 or 1, each column of the matrix represents the pairing result of the m conflicting bases. In the *j*-th pairing result, if *M*(*i*,*j*) = 1, it means the predicted result of Adaptive LSTM of *i*-th conflicting base is retained, if *M*(*i*,*j*) = 0, it means the predicted result of Adaptive LSTM of *i*-th conflicting base is not accepted and *S*(*i*) should be set as 0 to classify this base to unpaired base.

Correct(*M*[:,*j*]) is to correct the *j*-th pairing result according to the *j*-th column of *M*: *y’* is a copy of *y*, in the *j*-th pairing result, for each conflicting base, if *M*(*i,j*) = 1and the *y* (*Id*(*i*))-th base in primary sequence is conflicting base or unpaired base, keep *y’*(*Id*(i)) and set *y’*(*y* (*Id*(*i*))) = *Id*(*i*), else set *y’*(*Id*(*i*)) = 0.

Reducing unmatched pairs is to correct the predicted result according to the two conditions.

In Calculate_energy(*y’*), *E*(*j*) is the free energy of the *j*-th pairing result. And *M*_*best*_ is the structure with lowest free energy in *M* till now.

Change each pairing result: for each base of each pairing result, if *M*(*i,j*) = *M*_*best*_(*j*), set *M*(*i,j*) = 1-*M*(*i,j*) in a low possibility *p1*, else set *M*(*i,j*) = 1-*M*(*i,j*) in a high possibility *p2*.

### Evaluation metrics

Accuracy (ACC) is a commonly used evaluation metrics in classification. Sensitivity (SEN) and specificity (PPV) are commonly used in RNA secondary structure prediction [30]. Matthews correlation coefficient (MCC) is an evaluation metrics that combines sensitivity and specificity. This paper uses SEN, PPV, MCC and ACC to evaluate the model, they are calculated as follows:

3 $$ sen=\frac{TP}{TP+ FN} $$

4 $$ ppv=\frac{TP}{TP+ FP} $$

5 $$ mcc=\frac{TP\times TN- FP\times FN}{\sqrt{\left( TP+ FP\right)\left( TP+ FN\right)\left( TN+ FP\right)\left( TN+ FN\right)}} $$

6 $$ acc=\frac{TP+ TN}{TP+ TN+ FP+ FN} $$

Where *TP* (true positives) means the number of correctly predicted bases; *FN* (false negatives) means the number of bases that are not correctly predicted; *FP* (false negatives) means the number of unpaired bases that predicted to be paired; *TN* (true negatives) means the number of correctly predicted unpaired bases [31]. The range of *SEN*, *PPV* and *ACC* is between 0 and 1, while the range *MCC* is between − 1 and 1, and the higher these evaluation metrics are, the better the model is.

## Results and discussion

*Dataset.* The dataset of this paper comes from authoritative dataset RNA STRAND [32], including five subsets: TMR (The tmRNA website [33]),SPR (Sprinzl tRNA Database [34]),SRP (Signal recognition particle database [35]),RFA (The RNA family database [36])and ASE (RNase P Database [37]).There are 2493 sequences in the 5 datasets, the maximum and average length is 553 and 267.37 respectively. The number of sequences, the average sequence length, the minimum length and the maximum length are shown in Table 1. 90% of these sequences are randomly selected as training data and the rest 10% are testing data.

**Table 1:** Datasets

Dataset	Number	Average	Max	Min
TMR	721	361.1	463	102
ASE	454	332.6	486	189
SPR	622	77.3	93	54
SRP	383	224.7	533	66
RFA	313	118.9	553	40

### Comparison between adaptive-LSTM with and without energy-based filter

**Fig. 2:**
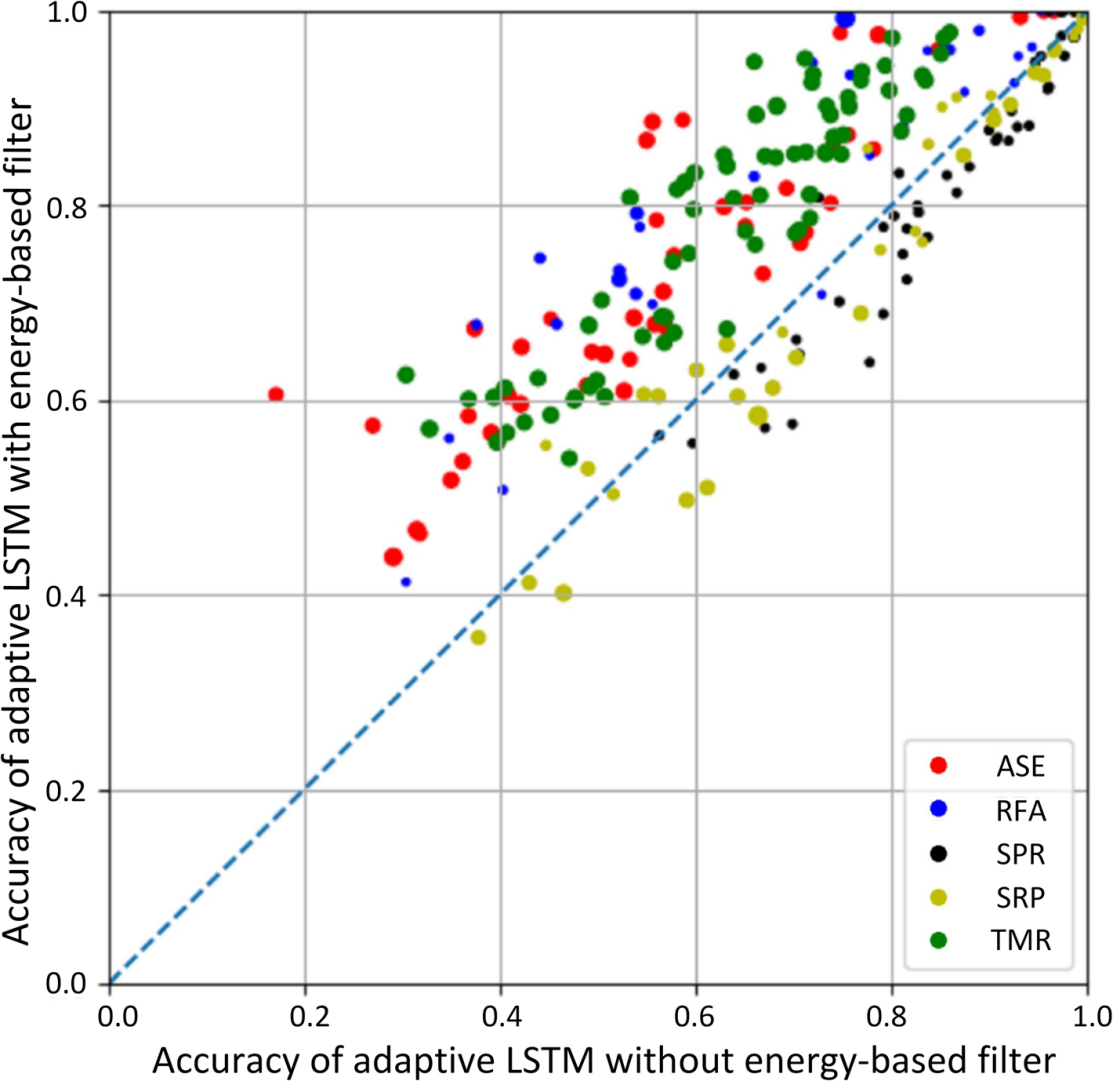
Scatter of accuracy comparison between adaptive-LSTM with and without filter

To prove the validity of the energy-based filter, a comparative experiment was carried out on the five datasets. Figure 2 shows the accuracy comparison of adaptive LSTM with energy-based filter(y-axis) and adaptive LSTM without energy-based filter(x-axis) on 249 test RNAs and the size of each points indicates the length of sequence. The number of points above the dotted line y = x are much more than the number of points under the dotted line. There are 172 RNAs (above the dotted line y = x) predicted by adaptive-LSTM with filter with higher accuracy than the accuracy predicted by adaptive-LSTM. On the dataset ASE and TMR adaptive-LSTM with filter predicted 100 and 100% of test RNAs are better than the prediction by adaptive-LSTM.

In the Fig. 2, most of the RNAs in dataset SPR (in black) with energy-based filter have lower accuracy than without the filter. There are two possible reasons. The first one is the average length of the sequences in SPR dataset is much shorter than other datasets and their folding structures are relatively simple. Less free energy gap of the simple structures leads to energy-based filter failure. The second possible reason is the percentage of bases with unknown base type is 11.44% on SPR dataset, while the percentage on TMR, ASE, SRP, RFA are 0.04, 0.02, 5.93%, which will cause inaccuracy of free-energy. Since the energy-base filter heavily relies on the free energy, the two reasons may make the energy-based filter failed over adaptive-LSTM.

#### Comparison between adaptive LSTM and other three classical methods

ProbKnot [38] assembles maximum expected accuracy structures from computed base-pairing probabilities in *O*(*N*^2^) time. Cylofold [39] is an RNA secondary structure prediction method with no algorithmic restriction in terms of pseudoknot complexity. Centroidfold [40] use novel estimators to maximize an objective function which is the weighted sum of the expected number of the true positives and that of the true negatives of the base pairs.

Comparison experiments between adaptive LSTM with energy-based filter and these three classical method was operated, the *SEN*, *PPV*, *ACC* and *MCC* of ProbKnot are 0.757, 0.587, 0.646 and 0.319, the *SEN*, ppv, *ACC* and *MCC* of Cylofold are 0.414, 0.319, 0.406 and 0.014, the *SEN*, *PPV*, *ACC* and *MCC* of Centroidfold are 0.673, 0.605, 0.654 and 0.307, the *SEN*, *PPV*, *ACC* and *MCC* of Adaptive LSTM are 0.927, 0.613, 0.689 and 0.483, the *SEN*, *PPV*, *ACC* and *MCC* of adaptive-LSTM with energy-based filter are 0.685, 0.883, 0.780 and 0.592.

Table 2 shows the *MCC* and *ACC* of the adaptive-LSTM (Adaptive), adaptive-LSTM with energy-based filter (Filter) and other classic RNA secondary structure-prediction methods. Adaptive LSTM is better than other three methods in all metrics and energy-based filter can further improve the *ACC* and *MCC*. Because the Cylofold algorithm does not allow for missing bases, it generated no results for SPR datasets in which the sequence information was incomplete.

**Table 2:** MCC and ACC of adaptive LSTM and other three methods

Dataset	Metrics	ProbKnot	Cylofold	CentroidFold	Adaptive	Filter
TMR	MCC	0.105	−0.043	0.106	0.434	**0.581**
	ACC	0.531	0.485	0.561	0.630	**0.786**
SPR	MCC	0.591	*	0.668	**0.786**	0.751
	ACC	0.796	*	0.834	**0.891**	0.870
SRP	MCC	0.262	−0.184	0.177	0.421	**0.475**
	ACC	0.613	0.396	0.584	**0.708**	0.690
RFA	MCC	0.398	0.256	0.299	**0.451**	0.699
	ACC	0.677	0.624	0.650	0.661	**0.834**
ASE	MCC	0.238	0.043	0.286	0.323	**0.484**
	ACC	0.611	0.523	0.642	0.556	**0.720**
Average	MCC	0.319	0.014	0.307	0.483	**0.592**
	ACC	0.646	0.406	0.654	0.689	**0.780**

Boldface represents the highest MCC or ACC in comparison with the other three methods

*indicates Cylofold does not generate results on SPR dataset, since Cylofold can not accept the sequence with missing bases in SPR dataset

Compared with the three classical methods, Adaptive LSTM have higher *ACC* in four datasets and higher MCC in all datasets, after adding the energy-based filter, the *MCC* is further improved to 0.78 on average. Because the Cylofold algorithm does not allow for missing bases, it generated no results for SPR datasets in which the sequence information was incomplete.

#### Case study on RNA with pseudoknots

**Fig. 3:**
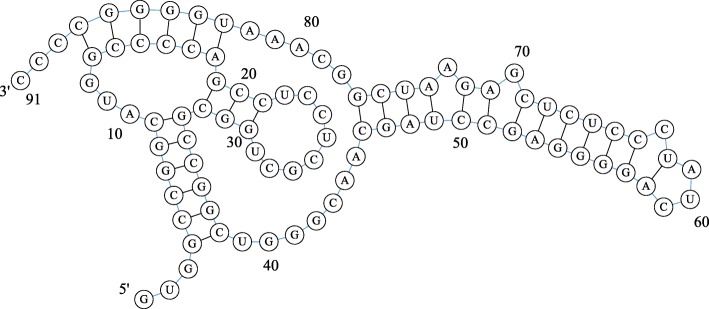
Native secondary structure of RFA_00633

RFA_00633 (hepatitis delta virus ribozyme) is an RNA sequence with pseudoknots and represents a noncoding RNA molecule found in the hepatitis delta virus that is necessary for viral replication and reportedly the only catalytic RNA required by a human pathogen for viability [41]. The length of its primary sequence is 91 bases, and its native secondary structure is shown in Fig. 3.

**Fig. 4:**
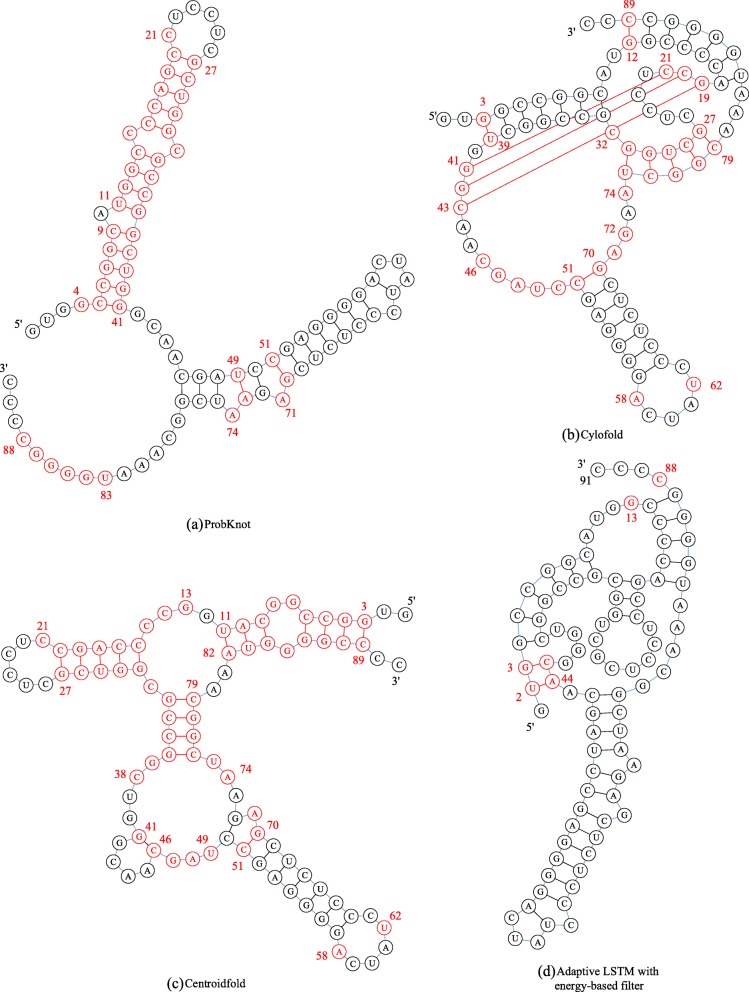
Predicted secondary structure of RFA_00633. **a** ProbKnot. **b** Cylofold. **c** Centroidfold. **d** Adaptive LSTM with energy-based filter

Figure 4 shows the predictive results from the ProbKnot, adaptive LSTM with energy-based filter, Centroidfold and Cylofold. There were no pseudoknots in the predicted structures, and the ACC values were 51.6 and 40.7% for ProbKnot and centroidfold, respectively Fig. 4a and c. Figure 4d shows the predicted structure with pseudoknots by cylofold, with an ACC of 63.7%.

Adaptive LSTM without energy-based filter cannot predict a valid result to construct a secondary structure because there are conflicting pairing bases. The structure predicted by the adaptive LSTM showed an *ACC* of 93.4%, which exceed that of the other three methods.

## Conclusions

This paper proposed a RNA secondary structure predicting method based on Adaptive LSTM with energy-based filter. This method addressed problems associated with truncating sequences in order to address problems associated with the variability in RNA-sequence length, with truncation often resulting in the loss of sequence information and incompleteness. Additionally, we added a dynamic weighting algorithm to alleviate problems related to the unbalanced distribution of samples and use energy-based filter to remove the conflicting pairing result. Experimental results showed that this method effectively improved the accuracy of RNA secondary structure prediction, as the *MCC* metrics were 16% higher than other 3 classical algorithms on average, and energy-based filter can further improve the *MCC* to 59.2% which is 28% higher than other methods. For tests using the TMR dataset harboring sequences with large span lengths, *MCC* and *ACC* values were 43.4 and 63%, respectively, which were 33 and 9.9% higher than that of the ProbKnot algorithm, respectively.

Our future research will focus on improving the network structure by adding convolutional layer or attention layer and use cross validation, which could further enhance the predictive accuracy of the model.

## Data Availability

Datasets, source codes and results can be access from http://eie.usts.edu.cn/prj/AdaptiveLSTMRNA/index.html.

## Abbreviations

A: Adenine
ACC: Accuracy
ASE: RNase P Database
C: Cytosine
FN: False negatives
FP: False positives
G: Ganciclovir
LSTM: Long short-term memory
MCC: Matthews correlation coefficient
NP: Non-deterministic polynomial
PPV: Specificity
RFA: The RNA family database
RNA STRAND: RNA secondary STRucture and statistical Analysis Database
RNA: Ribonucleic Acid
SEN: Sensitivity
SRP: Signal recognition particle database
TMR: The tmRNA website
TN: True negatives
TP: True positives
U: Uracil
